# 2,6-Diamino­pyridinium bis­(4-hydroxy­pyridine-2,6-dicarboxyl­ato-κ^3^ *O* ^2^,*N*,*O* ^6^)ferrate(III) dihydrate

**DOI:** 10.1107/S1600536808029280

**Published:** 2008-09-20

**Authors:** Masoud Rafizadeh, Zohreh Derikvand, Andya Nemati

**Affiliations:** a Faculty of Chemistry, Tarbiat Moallem University, Tehran, Iran

## Abstract

The reaction of iron(II) sulfate hepta­hydrate with the proton-transfer compound (pydaH)(hypydcH) (pyda = pyridine-2,6-diamine; hypydcH_2_ = 4-hydroxy­pyridine-2,6-dicarboxylic acid) in an aqueous solution led to the formation of the title compound, (C_5_H_8_N_3_)[Fe(C_7_H_3_NO_5_)_2_]·2H_2_O. The anion is a six-coordinated complex with a distorted octa­hedral geometry around the Fe^III^ atom. Extensive inter­molecular O—H⋯O, N—H⋯O and C—H⋯O hydrogen bonds, involving the complex anion, (pydaH)^+^ counter-ion and two uncoordinated water mol­ecules, and π–π [centroid-to-centroid distance 3.323 (11) Å] and C—O⋯π [O–centroid distance 3.150 (15) Å] inter­actions connect the various components into a supra­molecular structure.

## Related literature

For other complexes with pyridine­dicarboxylic acids, see: Rafizadeh *et al.* (2004 ▶, 2006 ▶, 2007*a* ▶,*b* ▶); Rafizadeh & Amani (2006 ▶).
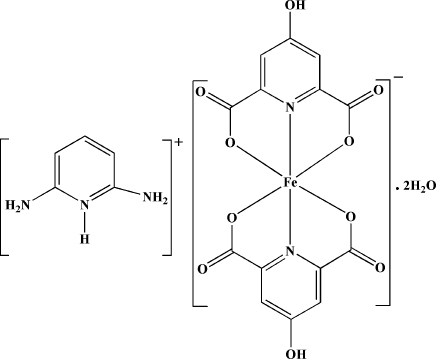


## Experimental

### 

#### Crystal data

(C_5_H_8_N_3_)[Fe(C_7_H_3_NO_5_)_2_]·2H_2_O
*M* *_r_* = 564.23
Monoclinic, 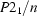

*a* = 6.9389 (4) Å
*b* = 20.8845 (12) Å
*c* = 14.9908 (8) Å
β = 96.371 (1)°
*V* = 2159.0 (2) Å^3^
*Z* = 4
Mo *K*α radiation
μ = 0.78 mm^−1^
*T* = 100 (2) K
0.40 × 0.40 × 0.20 mm

#### Data collection

Bruker SMART APEXII CCD area-detector diffractometer
Absorption correction: multi-scan (*SADABS*; Bruker, 2001 ▶) *T*_min_ = 0.746, *T*_max_ = 0.860
33555 measured reflections
8157 independent reflections
5648 reflections with *I* > 2σ(*I*)
*R*_int_ = 0.073

#### Refinement

*R*[*F* ^2^ > 2σ(*F* ^2^)] = 0.045
*wR*(*F* ^2^) = 0.110
*S* = 1.02
8157 reflections
342 parameters
H-atom parameters constrained
Δρ_max_ = 0.49 e Å^−3^
Δρ_min_ = −0.61 e Å^−3^

### 

Data collection: *APEX2* (Bruker, 2007 ▶); cell refinement: *SAINT* (Bruker, 2007 ▶); data reduction: *SAINT*; program(s) used to solve structure: *SHELXTL* (Sheldrick, 2008 ▶); program(s) used to refine structure: *SHELXTL*; molecular graphics: *SHELXTL*; software used to prepare material for publication: *SHELXTL*.

## Supplementary Material

Crystal structure: contains datablocks I. DOI: 10.1107/S1600536808029280/hy2152sup1.cif

Structure factors: contains datablocks I. DOI: 10.1107/S1600536808029280/hy2152Isup2.hkl

Additional supplementary materials:  crystallographic information; 3D view; checkCIF report

## Figures and Tables

**Table:** 

Fe1—O3	2.0101 (13)
Fe1—O8	2.0135 (14)
Fe1—N2	2.0392 (15)
Fe1—O6	2.0413 (13)
Fe1—N1	2.0478 (14)
Fe1—O1	2.0544 (13)

**Table:** 

O3—Fe1—O8	95.80 (6)
O3—Fe1—N2	107.52 (6)
O8—Fe1—N2	76.88 (6)
O3—Fe1—O6	91.49 (5)
O8—Fe1—O6	152.40 (5)
N2—Fe1—O6	75.54 (6)
O3—Fe1—N1	76.40 (5)
O8—Fe1—N1	105.50 (6)
N2—Fe1—N1	175.34 (6)
O6—Fe1—N1	102.08 (5)
O3—Fe1—O1	151.34 (5)
O8—Fe1—O1	94.61 (5)
N2—Fe1—O1	100.83 (5)
O6—Fe1—O1	91.53 (5)
N1—Fe1—O1	75.10 (5)

**Table 2:** Hydrogen-bond geometry (Å, °)

*D*—H⋯*A*	*D*—H	H⋯*A*	*D*⋯*A*	*D*—H⋯*A*
N3—H3*N*⋯O2^i^	0.92	2.00	2.8431 (19)	152
N4—H4*NA*⋯O2*W*^ii^	0.92	2.04	2.957 (2)	173
N4—H4*NB*⋯O3	0.92	2.33	3.139 (2)	147
O5—H5⋯O1*W*	0.85	1.74	2.566 (2)	164
O10—H10⋯O2*W*	0.85	1.80	2.614 (2)	159
N5—H5*NA*⋯O2^i^	0.92	1.98	2.800 (2)	148
N5—H5*NB*⋯O6^iii^	0.92	1.96	2.832 (2)	157
O1*W*—H1*WA*⋯O7^iv^	0.85	1.98	2.826 (2)	173
O1*W*—H1*WB*⋯O4^ii^	0.85	2.05	2.877 (2)	166
O2*W*—H2*WA*⋯O1^v^	0.85	1.88	2.716 (2)	168
O2*W*—H2*WB*⋯O9^vi^	0.85	1.87	2.709 (2)	168
C16—H16*A*⋯O3	0.95	2.55	3.323 (2)	139

Symmetry codes: (i) 
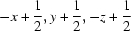
; (ii) 
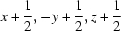
; (iii) 
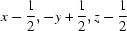
; (iv) 
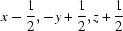
; (v) 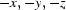; (vi) 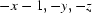.

**Fig. 1.**
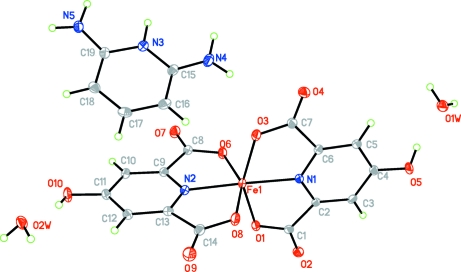
The molecular structure of the title compound. Displacement ellipsoids are drawn at the 50% probability level.

**Fig. 2.**
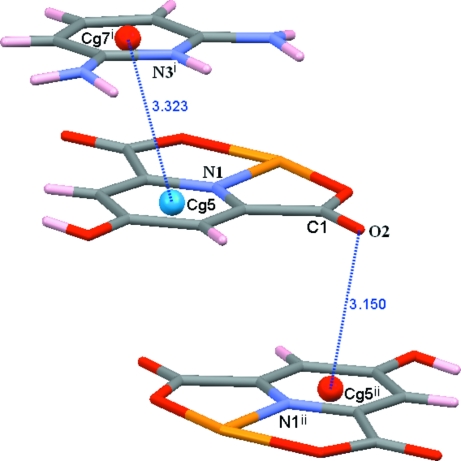
π–π Stacking interaction (Cg5···Cg7i) and and C—O···π interaction (C1—O2···Cg5ii) in the title compound. [Cg5: N1/C2–C6, Cg7: N3/C15–C19. Symmetry codes: (i) 1/2 + x, 1/2 - y, 1/2 + z; (ii) -x, -y, 1 - z.]

**Fig. 3.**
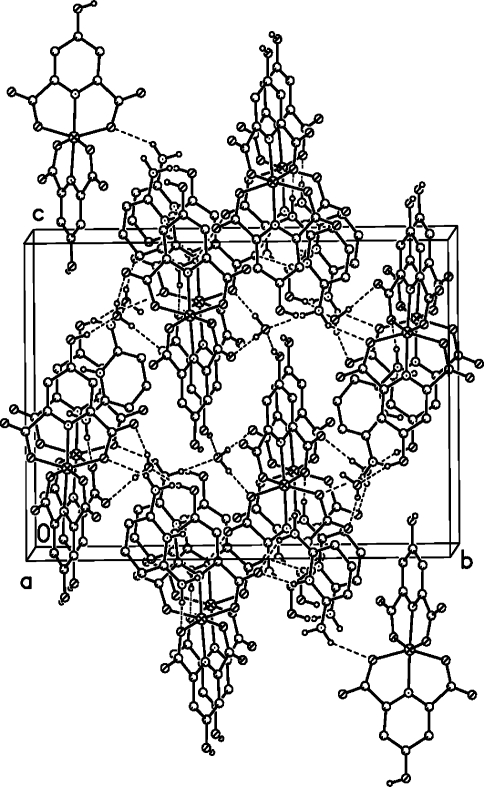
Crystal packing of the title compound. Hydrogen bonds are shown by dashed lines.

## supplementary crystallographic information

### Comment

Noncovalent interactions including hydrogen bonding, ion pairing, hydrophobic or hydrophilic and donor–acceptor interactions play a key role in chemical, catalytic and biochemical processes, as well as supramolecular chemistry and crystal engineering. Our research group has recently focused on synthesis of water soluble self-assembly systems that can function as suitable ligands in the synthesis of metal complexes. We have reported some complexes with pyridinedicarboxylic acids (Rafizadeh *et al.*, 2004, 2006, 2007*a*,*b*; Rafizadeh & Amani, 2006).

In the title compound (Fig. 1), the Fe^III^ atom has a distorted octahedral geometry. The bond angles (Table 1) and the torsion angles O6—Fe1—O1—C1 [100.80 (13)°], O1—Fe1—O6—C8 [103.59 (13)°], O8—Fe1—O3—C7 [104.38 (13)°] and O3—Fe1—O8—C14 [106.46 (14)°] indicate that two dianionic hypydc ligands are almost perpendicular to each other. In this work we used Fe^II^ ions as starting material. Most probably during the synthesis process, Fe^II^ was oxidized into Fe^III^. There are a large number of O—H···O, N—H···O and C—H···O hydrogen bonds between the cations, anions and water molecules (Table 2). Considerable π–π interaction [centroid–centroid distance = 3.323 (11) Å] between the cation and anion, and C—O···π interaction [O–centroid distance = 3.150 (15) Å] between two anions are observed (Fig. 2). Hydrogen bonds, π–π and C—O···π interactions result in the formation of a supramolecular structure (Fig. 3).

### Experimental

The reaction of FeSO_4_.7H_2_O (0.139 g, 0.5 mmol) in water (20 ml) with (pydaH)(hypydcH) (0.264 g, 1.0 mmol) in water (20 ml) gave colorless crystal of the title compound. Crystals suitable for X-ray diffraction were obtained by slow evaporation of the solvent at room temperature.

### Refinement

H atoms attached to O and N atoms and water molecules are located from difference Fourier maps and refined isotropically with their coordinates fixed. H atoms on C atoms were positioned geometrically and refined in riding model, with C—H = 0.95 Å and *U*_iso_(H) = 1.2*U*_eq_(C).

### Crystal data

**Table:**

(C_5_H_8_N_3_)[Fe(C_7_H_3_NO_5_)_2_]·2H_2_O	*F*(000) = 1156
*M**_r_* = 564.23	*D*_x_ = 1.736 Mg m^−^^3^
Monoclinic, *P*2_1_/*n*	Mo *K*α radiation, λ = 0.71073 Å
Hall symbol: -P 2yn	Cell parameters from 4719 reflections
*a* = 6.9389 (4) Å	θ = 2.4–31.9°
*b* = 20.8845 (12) Å	µ = 0.78 mm^−^^1^
*c* = 14.9908 (8) Å	*T* = 100 K
β = 96.371 (1)°	Prism, colourless
*V* = 2159.0 (2) Å^3^	0.40 × 0.40 × 0.20 mm
*Z* = 4	

### Data collection

**Table:**

Bruker SMART APEXII CCD area-detector diffractometer	8157 independent reflections
Radiation source: fine-focus sealed tube	5648 reflections with *I* > 2σ(*I*)
graphite	*R*_int_ = 0.073
φ and ω scans	θ_max_ = 33.1°, θ_min_ = 1.7°
Absorption correction: multi-scan (SADABS; Bruker, 2001)	*h* = −10→10
*T*_min_ = 0.746, *T*_max_ = 0.860	*k* = −31→32
33555 measured reflections	*l* = −22→22

### Refinement

**Table:**

Refinement on *F*^2^	Primary atom site location: structure-invariant direct methods
Least-squares matrix: full	Secondary atom site location: difference Fourier map
*R*[*F*^2^ > 2σ(*F*^2^)] = 0.045	Hydrogen site location: mixed
*wR*(*F*^2^) = 0.110	H-atom parameters constrained
*S* = 1.02	*w* = 1/[σ^2^(*F*_o_^2^) + (0.0425*P*)^2^ + 0.6357*P*] where *P* = (*F*_o_^2^ + 2*F*_c_^2^)/3
8157 reflections	(Δ/σ)_max_ = 0.001
342 parameters	Δρ_max_ = 0.49 e Å^−^^3^
0 restraints	Δρ_min_ = −0.61 e Å^−^^3^

### Special details

**Table:**

Geometry. All e.s.d.'s (except the e.s.d. in the dihedral angle between two l.s. planes) are estimated using the full covariance matrix. The cell e.s.d.'s are taken into account individually in the estimation of e.s.d.'s in distances, angles and torsion angles; correlations between e.s.d.'s in cell parameters are only used when they are defined by crystal symmetry. An approximate (isotropic) treatment of cell e.s.d.'s is used for estimating e.s.d.'s involving l.s. planes.

### Fractional atomic coordinates and isotropic or equivalent isotropic displacement parameters (Å^2^)

**Table:**

	*x*	*y*	*z*	*U*_iso_*/*U*_eq_	
Fe1	0.03345 (4)	0.098201 (12)	0.291874 (16)	0.01147 (7)	
O1	0.18095 (19)	0.01628 (6)	0.33338 (8)	0.0138 (3)	
O2	0.31460 (19)	−0.04217 (6)	0.44867 (9)	0.0154 (3)	
O3	−0.08559 (19)	0.18304 (6)	0.31759 (8)	0.0147 (3)	
O4	−0.1601 (2)	0.25261 (6)	0.42183 (9)	0.0164 (3)	
O5	0.1267 (2)	0.11113 (7)	0.70162 (8)	0.0179 (3)	
H5	0.0929	0.1486	0.7156	0.041 (8)*	
N1	0.0599 (2)	0.10213 (7)	0.42918 (9)	0.0104 (3)	
C1	0.2245 (3)	0.00489 (8)	0.41778 (12)	0.0119 (3)	
C2	0.1537 (3)	0.05567 (8)	0.47787 (11)	0.0105 (3)	
C3	0.1796 (3)	0.05772 (8)	0.56992 (11)	0.0120 (3)	
H3A	0.2470	0.0246	0.6037	0.014*	
C4	0.1027 (3)	0.11076 (8)	0.61299 (12)	0.0120 (3)	
C5	0.0084 (3)	0.15997 (8)	0.56065 (12)	0.0119 (3)	
H5A	−0.0422	0.1965	0.5879	0.014*	
C6	−0.0080 (3)	0.15336 (8)	0.46877 (11)	0.0110 (3)	
C7	−0.0940 (3)	0.20139 (9)	0.39982 (12)	0.0128 (3)	
O6	0.28312 (19)	0.14151 (6)	0.26334 (8)	0.0149 (3)	
O7	0.4605 (2)	0.17585 (7)	0.15648 (9)	0.0187 (3)	
O8	−0.2194 (2)	0.05293 (6)	0.25578 (8)	0.0158 (3)	
O9	−0.4188 (2)	0.01213 (7)	0.14115 (9)	0.0205 (3)	
O10	−0.0042 (2)	0.09000 (7)	−0.11598 (9)	0.0201 (3)	
H10	−0.1192	0.0795	−0.1361	0.039 (8)*	
N2	0.0277 (2)	0.09017 (7)	0.15604 (10)	0.0117 (3)	
C8	0.3215 (3)	0.14715 (9)	0.18084 (12)	0.0133 (3)	
C9	0.1707 (3)	0.11574 (8)	0.11447 (12)	0.0120 (3)	
C10	0.1645 (3)	0.11537 (9)	0.02270 (12)	0.0142 (3)	
H10A	0.2677	0.1328	−0.0063	0.017*	
C11	0.0007 (3)	0.08840 (9)	−0.02725 (12)	0.0146 (4)	
C12	−0.1484 (3)	0.06195 (9)	0.01761 (12)	0.0140 (3)	
H12A	−0.2596	0.0432	−0.0148	0.017*	
C13	−0.1279 (3)	0.06408 (9)	0.11011 (12)	0.0129 (3)	
C14	−0.2708 (3)	0.04000 (9)	0.17213 (12)	0.0144 (3)	
N3	0.0302 (2)	0.33319 (7)	0.07019 (10)	0.0133 (3)	
H3N	0.0921	0.3714	0.0844	0.028 (7)*	
N4	0.0433 (3)	0.30671 (8)	0.22110 (11)	0.0195 (3)	
H4NA	0.0914	0.3465	0.2380	0.045 (8)*	
H4NB	−0.0055	0.2836	0.2658	0.051 (9)*	
N5	0.0395 (2)	0.36729 (8)	−0.07645 (10)	0.0154 (3)	
H5NA	0.0937	0.4051	−0.0543	0.029 (7)*	
H5NB	−0.0126	0.3660	−0.1356	0.050 (9)*	
C15	−0.0200 (3)	0.29254 (9)	0.13517 (12)	0.0140 (3)	
C16	−0.1329 (3)	0.23951 (9)	0.10904 (13)	0.0160 (4)	
H16A	−0.1734	0.2109	0.1526	0.019*	
C17	−0.1855 (3)	0.22903 (9)	0.01850 (13)	0.0164 (4)	
H17A	−0.2638	0.1929	0.0006	0.020*	
C18	−0.1282 (3)	0.26914 (9)	−0.04682 (13)	0.0151 (4)	
H18A	−0.1615	0.2601	−0.1087	0.018*	
C19	−0.01989 (16)	0.32341 (5)	−0.01954 (7)	0.0129 (3)	
O1W	0.05579 (16)	0.21932 (5)	0.77225 (7)	0.0176 (3)	
H1WA	0.0359	0.2525	0.7396	0.042 (8)*	
H1WB	0.1458	0.2209	0.8155	0.057 (10)*	
O2W	−0.3280 (2)	0.06443 (7)	−0.21595 (9)	0.0173 (3)	
H2WA	−0.2977	0.0393	−0.2567	0.045 (8)*	
H2WB	−0.4203	0.0440	−0.1961	0.066 (11)*	

### Atomic displacement parameters (Å^2^)

**Table:**

	*U*^11^	*U*^22^	*U*^33^	*U*^12^	*U*^13^	*U*^23^
Fe1	0.01484 (13)	0.01232 (12)	0.00737 (11)	−0.00036 (10)	0.00172 (9)	0.00067 (9)
O1	0.0191 (7)	0.0131 (6)	0.0095 (6)	0.0017 (5)	0.0023 (5)	−0.0006 (5)
O2	0.0174 (7)	0.0130 (6)	0.0155 (6)	0.0031 (5)	0.0001 (5)	0.0004 (5)
O3	0.0197 (7)	0.0144 (6)	0.0101 (6)	0.0022 (5)	0.0022 (5)	0.0020 (5)
O4	0.0204 (7)	0.0131 (6)	0.0153 (6)	0.0033 (5)	0.0006 (5)	−0.0005 (5)
O5	0.0292 (8)	0.0171 (7)	0.0073 (6)	0.0023 (6)	0.0018 (5)	−0.0009 (5)
N1	0.0120 (7)	0.0103 (7)	0.0089 (6)	−0.0010 (5)	0.0013 (5)	−0.0004 (5)
C1	0.0124 (8)	0.0108 (8)	0.0129 (8)	−0.0026 (6)	0.0026 (6)	−0.0005 (6)
C2	0.0115 (8)	0.0097 (8)	0.0103 (8)	−0.0015 (6)	0.0016 (6)	0.0010 (6)
C3	0.0135 (8)	0.0110 (8)	0.0113 (8)	−0.0023 (6)	0.0010 (6)	0.0026 (6)
C4	0.0130 (8)	0.0130 (8)	0.0099 (7)	−0.0021 (6)	0.0006 (6)	−0.0007 (6)
C5	0.0137 (8)	0.0106 (8)	0.0119 (8)	−0.0021 (6)	0.0032 (6)	−0.0004 (6)
C6	0.0111 (8)	0.0113 (8)	0.0107 (8)	−0.0006 (6)	0.0016 (6)	0.0010 (6)
C7	0.0131 (8)	0.0130 (8)	0.0124 (8)	−0.0009 (7)	0.0023 (6)	0.0000 (6)
O6	0.0163 (7)	0.0183 (7)	0.0099 (6)	−0.0033 (5)	0.0011 (5)	0.0001 (5)
O7	0.0185 (7)	0.0222 (7)	0.0157 (6)	−0.0068 (6)	0.0034 (5)	0.0009 (5)
O8	0.0178 (7)	0.0201 (7)	0.0099 (6)	−0.0022 (5)	0.0024 (5)	0.0018 (5)
O9	0.0177 (7)	0.0250 (8)	0.0184 (7)	−0.0080 (6)	0.0001 (5)	0.0000 (6)
O10	0.0235 (8)	0.0282 (8)	0.0083 (6)	−0.0037 (6)	0.0009 (5)	−0.0014 (5)
N2	0.0138 (7)	0.0113 (7)	0.0101 (7)	−0.0003 (6)	0.0013 (5)	0.0008 (5)
C8	0.0163 (9)	0.0120 (8)	0.0114 (8)	−0.0002 (7)	0.0006 (6)	0.0005 (6)
C9	0.0140 (8)	0.0109 (8)	0.0116 (8)	−0.0003 (6)	0.0035 (6)	0.0005 (6)
C10	0.0181 (9)	0.0140 (8)	0.0110 (8)	−0.0009 (7)	0.0041 (7)	0.0008 (6)
C11	0.0208 (9)	0.0130 (8)	0.0102 (8)	0.0013 (7)	0.0024 (7)	−0.0011 (6)
C12	0.0172 (9)	0.0127 (8)	0.0117 (8)	−0.0010 (7)	−0.0011 (7)	−0.0008 (6)
C13	0.0158 (9)	0.0107 (8)	0.0124 (8)	−0.0005 (7)	0.0022 (7)	−0.0001 (6)
C14	0.0156 (9)	0.0138 (8)	0.0137 (8)	0.0003 (7)	0.0010 (7)	0.0020 (6)
N3	0.0132 (7)	0.0133 (7)	0.0132 (7)	0.0000 (6)	0.0012 (6)	0.0014 (6)
N4	0.0245 (9)	0.0203 (9)	0.0135 (8)	−0.0020 (7)	0.0012 (6)	0.0044 (6)
N5	0.0178 (8)	0.0167 (8)	0.0114 (7)	−0.0017 (6)	0.0010 (6)	−0.0002 (6)
C15	0.0133 (9)	0.0142 (8)	0.0147 (8)	0.0019 (7)	0.0025 (7)	0.0027 (7)
C16	0.0158 (9)	0.0126 (8)	0.0201 (9)	0.0023 (7)	0.0034 (7)	0.0036 (7)
C17	0.0144 (9)	0.0114 (8)	0.0234 (10)	0.0019 (7)	0.0020 (7)	−0.0021 (7)
C18	0.0153 (9)	0.0146 (9)	0.0155 (8)	0.0020 (7)	0.0023 (7)	−0.0023 (7)
C19	0.0108 (8)	0.0153 (8)	0.0128 (8)	0.0037 (7)	0.0021 (6)	−0.0002 (6)
O1W	0.0221 (7)	0.0167 (7)	0.0134 (6)	0.0014 (6)	−0.0011 (5)	−0.0008 (5)
O2W	0.0201 (7)	0.0194 (7)	0.0129 (6)	−0.0013 (6)	0.0041 (5)	−0.0039 (5)

### Geometric parameters (Å, °)

**Table:**

Fe1—O3	2.0101 (13)	N2—C9	1.340 (2)
Fe1—O8	2.0135 (14)	C8—C9	1.511 (3)
Fe1—N2	2.0392 (15)	C9—C10	1.372 (2)
Fe1—O6	2.0413 (13)	C10—C11	1.407 (3)
Fe1—N1	2.0478 (14)	C10—H10A	0.9500
Fe1—O1	2.0544 (13)	C11—C12	1.408 (3)
O1—C1	1.290 (2)	C12—C13	1.379 (2)
O2—C1	1.227 (2)	C12—H12A	0.9500
O3—C7	1.298 (2)	C13—C14	1.518 (3)
O4—C7	1.224 (2)	N3—C15	1.366 (2)
O5—C4	1.321 (2)	N3—C19	1.3668 (18)
O5—H5	0.8501	N3—H3N	0.9200
N1—C6	1.335 (2)	N4—C15	1.347 (2)
N1—C2	1.339 (2)	N4—H4NA	0.9200
C1—C2	1.509 (2)	N4—H4NB	0.9200
C2—C3	1.372 (2)	N5—C19	1.3475 (19)
C3—C4	1.416 (2)	N5—H5NA	0.9200
C3—H3A	0.9500	N5—H5NB	0.9199
C4—C5	1.409 (2)	C15—C16	1.388 (3)
C5—C6	1.376 (2)	C16—C17	1.383 (3)
C5—H5A	0.9500	C16—H16A	0.9500
C6—C7	1.514 (2)	C17—C18	1.380 (3)
O6—C8	1.299 (2)	C17—H17A	0.9500
O7—C8	1.225 (2)	C18—C19	1.396 (2)
O8—C14	1.294 (2)	C18—H18A	0.9500
O9—C14	1.226 (2)	O1W—H1WA	0.8499
O10—C11	1.327 (2)	O1W—H1WB	0.8500
O10—H10	0.8500	O2W—H2WA	0.8500
N2—C13	1.331 (2)	O2W—H2WB	0.8499
			
O3—Fe1—O8	95.80 (6)	O7—C8—O6	125.40 (17)
O3—Fe1—N2	107.52 (6)	O7—C8—C9	121.77 (16)
O8—Fe1—N2	76.88 (6)	O6—C8—C9	112.80 (15)
O3—Fe1—O6	91.49 (5)	N2—C9—C10	121.45 (17)
O8—Fe1—O6	152.40 (5)	N2—C9—C8	111.18 (15)
N2—Fe1—O6	75.54 (6)	C10—C9—C8	127.24 (16)
O3—Fe1—N1	76.40 (5)	C9—C10—C11	117.99 (17)
O8—Fe1—N1	105.50 (6)	C9—C10—H10A	121.0
N2—Fe1—N1	175.34 (6)	C11—C10—H10A	121.0
O6—Fe1—N1	102.08 (5)	O10—C11—C10	116.88 (17)
O3—Fe1—O1	151.34 (5)	O10—C11—C12	123.40 (17)
O8—Fe1—O1	94.61 (5)	C10—C11—C12	119.72 (16)
N2—Fe1—O1	100.83 (5)	C13—C12—C11	117.96 (17)
O6—Fe1—O1	91.53 (5)	C13—C12—H12A	121.0
N1—Fe1—O1	75.10 (5)	C11—C12—H12A	121.0
C1—O1—Fe1	120.35 (11)	N2—C13—C12	121.36 (17)
C7—O3—Fe1	120.33 (11)	N2—C13—C14	111.48 (15)
C4—O5—H5	104.2	C12—C13—C14	127.15 (17)
C6—N1—C2	120.86 (15)	O9—C14—O8	126.56 (17)
C6—N1—Fe1	118.84 (12)	O9—C14—C13	120.12 (16)
C2—N1—Fe1	120.19 (12)	O8—C14—C13	113.32 (16)
O2—C1—O1	124.88 (16)	C15—N3—C19	123.48 (15)
O2—C1—C2	121.58 (16)	C15—N3—H3N	121.5
O1—C1—C2	113.54 (15)	C19—N3—H3N	114.8
N1—C2—C3	121.80 (16)	C15—N4—H4NA	122.1
N1—C2—C1	110.79 (14)	C15—N4—H4NB	118.2
C3—C2—C1	127.40 (16)	H4NA—N4—H4NB	115.2
C2—C3—C4	117.97 (16)	C19—N5—H5NA	119.7
C2—C3—H3A	121.0	C19—N5—H5NB	118.4
C4—C3—H3A	121.0	H5NA—N5—H5NB	118.3
O5—C4—C5	123.70 (16)	N4—C15—N3	117.61 (17)
O5—C4—C3	116.84 (16)	N4—C15—C16	124.00 (17)
C5—C4—C3	119.46 (16)	N3—C15—C16	118.39 (17)
C6—C5—C4	117.76 (16)	C17—C16—C15	118.84 (17)
C6—C5—H5A	121.1	C17—C16—H16A	120.6
C4—C5—H5A	121.1	C15—C16—H16A	120.6
N1—C6—C5	122.10 (16)	C18—C17—C16	122.33 (18)
N1—C6—C7	111.05 (14)	C18—C17—H17A	118.8
C5—C6—C7	126.81 (16)	C16—C17—H17A	118.8
O4—C7—O3	124.87 (17)	C17—C18—C19	118.23 (16)
O4—C7—C6	121.74 (16)	C17—C18—H18A	120.9
O3—C7—C6	113.38 (15)	C19—C18—H18A	120.9
C8—O6—Fe1	120.52 (12)	N5—C19—N3	117.33 (12)
C14—O8—Fe1	119.79 (12)	N5—C19—C18	124.01 (13)
C11—O10—H10	105.5	N3—C19—C18	118.66 (13)
C13—N2—C9	121.52 (15)	H1WA—O1W—H1WB	117.8
C13—N2—Fe1	118.37 (12)	H2WA—O2W—H2WB	101.4
C9—N2—Fe1	119.87 (12)		
			
O3—Fe1—O1—C1	4.9 (2)	O3—Fe1—O8—C14	106.46 (14)
O8—Fe1—O1—C1	−106.13 (13)	N2—Fe1—O8—C14	−0.21 (13)
N2—Fe1—O1—C1	176.37 (13)	O6—Fe1—O8—C14	1.9 (2)
O6—Fe1—O1—C1	100.80 (13)	N1—Fe1—O8—C14	−176.08 (13)
N1—Fe1—O1—C1	−1.29 (13)	O1—Fe1—O8—C14	−100.28 (14)
O8—Fe1—O3—C7	104.38 (13)	O3—Fe1—N2—C13	−89.32 (14)
N2—Fe1—O3—C7	−177.58 (13)	O8—Fe1—N2—C13	2.70 (13)
O6—Fe1—O3—C7	−102.28 (13)	O6—Fe1—N2—C13	−176.26 (14)
N1—Fe1—O3—C7	−0.21 (13)	O1—Fe1—N2—C13	94.96 (14)
O1—Fe1—O3—C7	−6.4 (2)	O3—Fe1—N2—C9	85.10 (14)
O3—Fe1—N1—C6	0.52 (13)	O8—Fe1—N2—C9	177.12 (15)
O8—Fe1—N1—C6	−91.82 (14)	O6—Fe1—N2—C9	−1.85 (13)
O6—Fe1—N1—C6	89.12 (14)	O1—Fe1—N2—C9	−90.63 (14)
O1—Fe1—N1—C6	177.47 (14)	Fe1—O6—C8—O7	175.02 (15)
O3—Fe1—N1—C2	−175.72 (14)	Fe1—O6—C8—C9	−3.1 (2)
O8—Fe1—N1—C2	91.94 (14)	C13—N2—C9—C10	−0.9 (3)
O6—Fe1—N1—C2	−87.13 (14)	Fe1—N2—C9—C10	−175.18 (13)
O1—Fe1—N1—C2	1.22 (13)	C13—N2—C9—C8	175.08 (16)
Fe1—O1—C1—O2	−178.87 (14)	Fe1—N2—C9—C8	0.8 (2)
Fe1—O1—C1—C2	1.16 (19)	O7—C8—C9—N2	−176.81 (17)
C6—N1—C2—C3	1.7 (3)	O6—C8—C9—N2	1.4 (2)
Fe1—N1—C2—C3	177.91 (13)	O7—C8—C9—C10	−1.1 (3)
C6—N1—C2—C1	−177.17 (15)	O6—C8—C9—C10	177.16 (17)
Fe1—N1—C2—C1	−1.00 (19)	N2—C9—C10—C11	1.3 (3)
O2—C1—C2—N1	179.93 (16)	C8—C9—C10—C11	−174.01 (17)
O1—C1—C2—N1	−0.1 (2)	C9—C10—C11—C12	−1.1 (3)
O2—C1—C2—C3	1.1 (3)	O10—C11—C12—C13	−178.96 (17)
O1—C1—C2—C3	−178.93 (17)	C10—C11—C12—C13	0.5 (3)
N1—C2—C3—C4	0.3 (3)	C9—N2—C13—C12	0.3 (3)
C1—C2—C3—C4	179.06 (17)	Fe1—N2—C13—C12	174.63 (13)
C2—C3—C4—O5	178.92 (16)	C9—N2—C13—C14	−178.61 (16)
C2—C3—C4—C5	−1.8 (3)	Fe1—N2—C13—C14	−4.3 (2)
O5—C4—C5—C6	−179.53 (17)	C11—C12—C13—N2	−0.1 (3)
C3—C4—C5—C6	1.3 (3)	C11—C12—C13—C14	178.62 (17)
C2—N1—C6—C5	−2.4 (3)	Fe1—O8—C14—O9	179.03 (15)
Fe1—N1—C6—C5	−178.57 (13)	Fe1—O8—C14—C13	−1.9 (2)
C2—N1—C6—C7	175.52 (15)	N2—C13—C14—O9	−176.93 (17)
Fe1—N1—C6—C7	−0.69 (19)	C12—C13—C14—O9	4.2 (3)
C4—C5—C6—N1	0.8 (3)	N2—C13—C14—O8	3.9 (2)
C4—C5—C6—C7	−176.72 (17)	C12—C13—C14—O8	−174.90 (18)
Fe1—O3—C7—O4	178.48 (14)	C19—N3—C15—N4	178.12 (15)
Fe1—O3—C7—C6	−0.1 (2)	C19—N3—C15—C16	−2.1 (3)
N1—C6—C7—O4	−178.12 (17)	N4—C15—C16—C17	−178.46 (18)
C5—C6—C7—O4	−0.4 (3)	N3—C15—C16—C17	1.7 (3)
N1—C6—C7—O3	0.5 (2)	C15—C16—C17—C18	0.6 (3)
C5—C6—C7—O3	178.25 (17)	C16—C17—C18—C19	−2.6 (3)
O3—Fe1—O6—C8	−104.91 (13)	C15—N3—C19—N5	−179.87 (16)
O8—Fe1—O6—C8	0.6 (2)	C15—N3—C19—C18	0.0 (2)
N2—Fe1—O6—C8	2.80 (13)	C17—C18—C19—N5	−177.84 (16)
N1—Fe1—O6—C8	178.68 (13)	C17—C18—C19—N3	2.3 (2)
O1—Fe1—O6—C8	103.59 (13)		

### Hydrogen-bond geometry (Å, °)

**Table:**

*D*—H···*A*	*D*—H	H···*A*	*D*···*A*	*D*—H···*A*
N3—H3N···O2^i^	0.92	2.00	2.8431 (19)	152
N4—H4NA···O2W^ii^	0.92	2.04	2.957 (2)	173
N4—H4NB···O3	0.92	2.33	3.139 (2)	147
O5—H5···O1W	0.85	1.74	2.566 (2)	164
O10—H10···O2W	0.85	1.80	2.614 (2)	159
N5—H5NA···O2^i^	0.92	1.98	2.800 (2)	148
N5—H5NB···O6^iii^	0.92	1.96	2.832 (2)	157
O1W—H1WA···O7^iv^	0.85	1.98	2.826 (2)	173
O1W—H1WB···O4^ii^	0.85	2.05	2.877 (2)	166
O2W—H2WA···O1^v^	0.85	1.88	2.716 (2)	168
O2W—H2WB···O9^vi^	0.85	1.87	2.709 (2)	168
C16—H16A···O3	0.95	2.55	3.323 (2)	139

Symmetry codes: (i) −*x*+1/2, *y*+1/2, −*z*+1/2; (ii) *x*+1/2, −*y*+1/2, *z*+1/2; (iii) *x*−1/2, −*y*+1/2, *z*−1/2; (iv) *x*−1/2, −*y*+1/2, *z*+1/2; (v) −*x*, −*y*, −*z*; (vi) −*x*−1, −*y*, −*z*.
